# Astaxanthin as a Potential Antioxidant to Improve Health and Production Performance of Broiler Chicken

**DOI:** 10.1155/2022/4919442

**Published:** 2022-04-14

**Authors:** Herinda Pertiwi, Mohamad Yusril Nur Mahendra, Juriah Kamaludeen

**Affiliations:** ^1^Department of Health Studies, Faculty of Vocational Studies Airlangga University, Jalan Dharmawangsa Dalam 28-30, Surabaya 60286, Indonesia; ^2^Department of Animal Science and Fishery, Universiti Putra Malaysia, Bintulu Serawak Campus, Nyabau Road 97008, Serawak, Malaysia

## Abstract

Recent interest in carotenoids has increased due to their antioxidant and production performance. Astaxanthin (AST) is a xanthophyll carotenoid abundantly distributed in microalgae, which is described as a highly potent antioxidant. Therefore, recent studies have tended to investigate the role of antioxidants in improving metabolic processes and physiological functioning of the body. It is now evident that AST could significantly reduce free radicals and oxidative stress and help to maintain a healthy state. Moreover, AST also could improve the performance of broiler chicken by increasing the daily feed intake, followed by improvement in the food conversion rate.

## 1. Introduction

Poultry meat is considered one of the most popular sources of animal protein with a high nutritional value and healthy ingredients for the people of the whole world due to their biological importance in cell regeneration and maintaining human health [1]. The poultry industry is one of the important elements in a country to fulfill animal protein demand, and researchers have made their efforts to contribute to their development by increasing production and health efficiency and eliminating problems that are exposed to them, including oxidative stress stimulated by heat stress [2].

Oxidative stress is one of the most important reasons that stimulate a decrease in growth performance, deterioration of immunity, and high mortality rate, which initiates economic losses. Therefore, the free radical formation of reactive oxygen species (ROS) must be improved [3]. Under high ambient temperature conditions, ROS generation increases in various body tissues as the heat load elevates [4]. Oxidative reactions also could be increased by heat stress, which then affects the meat quality by affecting meat color, ultimate pH, meat tenderness, and water-holding capacity [5].

Recent research and studies have tended to find solutions and alternatives to reduce the oxidative stress effect by using natural additives such as carotenoids in poultry diets as an alternative [6]. Carotenoids from *Xanthophyllomyces dendrorhous* have been used in the poultry industry for many years to pigment eggs and meat [7]. Astaxanthin (AST) is one of a group of natural pigments, known as xanthophyll carotenoids, which exhibit a wide variety of biological activities [8]. AST has a wide range of applications in the food, feed, cosmetics, aquaculture, nutraceutical, and pharmaceutical industries due to its free radical scavenging capacity [9].

The primary advantage of AST is its high capacity to capture free radicals and the ROS found in biological systems [10]. With the known antioxidant activities, AST from *Haematococcus pluvialis* also might be used to protect animals from heat-stress-mediated oxidative insults [11]. In addition, studies have shown that AST from freeze-dried *Phaffia* yeast also has several other biological activities, including anticancer, anti-inflammatory, and antidiabetic effects. Furthermore, AST from freeze-dried *Phaffia* yeast was 5.0 mg/g yeast having beneficial effects on the skin, reproduction, and blood pressure [12]. However, until now, only a limited brief review has discussed AST application in animals, particularly broiler chicken performance (Table 1). Therefore, this article focused on the potential antioxidant role of AST to improve health and production performance and also reviewed dietary sources, dietary intake, bioavailability, absorption, distribution, and heat (oxidative stress) protective effects in broiler chicken.

## 2. Data Collection

Information about AST was retrieved from a literature search of electronic databases such as PubMed, Elsevier, ResearchGate, Academia, and Google Scholar. The keywords used to perform the search were Astaxanthin (AST) in broiler chicken, antioxidant, and AST health protective.

## 3. Astaxanthin Dietary Sources

There are 2 sources of astaxanthin (AST), namely, biological acquisition and artificial synthesis. Natural AST has a *trans* structure that has relatively stable activity, while synthetic AST has a *cis* structure that has low bioavailability [27]. Natural sources of AST are generally found in a wide variety of plants, algae, and kinds of seafood [12].

AST extracted from *H. pluvialis* could be used as a safe natural antioxidant, approved by the European Food and Safety Authority (EFSA) and the Food and Allergy Committee (NDA), which showed safety and suitability as a nutritional supplement for humans and animals [13, 22]. AST could accumulate in *H. pluvialis* up to 5%, which makes it a major source of carotenoid pigments [23]. *H. pluvialis* is unicellular microalgae found in many habitats and is used as the main microorganism to produce AST [9]. The main form of AST present in *H. pluvialis* is monoester [24]. AST synthesis in *H. pluvialis* occurs in response to high salinity, stress due to high light intensity, nitrogen or phosphorus deficits, and the presence of salicylic acid and ethanol [25]. Moreover, AST synthesis in *H. pluvialis* is directly correlated in space and time with the deposition of cellular reserves in lipid droplets under conditions of cellular stress [26]. Meanwhile, the key to AST biosynthesis in *H. pluvialis* is through the nonmevalonate (MEP) pathway which belongs to the isopentenyl pyrophosphate (IPP) pathway [9].

Before *H. pluvialis* became commercially available, natural sources of AST included shrimp, salmon, crabs, shellfish, trout, red sea bream, lobster, fish eggs, krill, crawfish oil, some types of phytoplankton like *Chlorella zofingiensis*, *Chlorococcum* sp., bacteria like *Mycobacterium lacticola*, *Brevibacterium*, *Agrobacterium aurantiacum*, *Alcaligenes* sp. strain PC-1, *Paracoccus carotinifaciens*, and also yeasts such as *Phaffia yeast* [24, 27–29]. *Phaffia rhodozyma*, also known as red yeast, which has changed its name to *X. dendrorhous*, contains 0.3% AST [13]. However, other studies reveal that AST concentrations in *Phaffia* yeast are ranging from 0.15% to 0.40% in the oils. Meanwhile, *H. pluvialis* contains between 1.5% and 3.0% AST [8].

In the world market scale, the need for AST is mostly met through the synthesis of AST compared to natural pigment sources, but attention to alternative sources of AST from microorganisms in recent years has increased, especially in the types of *H. pluvialis* and *P. rhodozyma* which are useful for the production of AST on an industrial scale [13]. *H. pluvialis* has a carotenoid response that makes it able to survive in an unfavorable environment, making it a source of AST that is commonly used on an industrial scale [26]. However, the production of AST from *H. pluvialis* is quite expensive due to the fact that it requires a high-capacity photobioreactor growth medium, whereas its growth was slow when using traditional media exposed to H. pluvialis, and it would produce AST which is protected by thick-walled immobile hematocysts [25, 30].

Production of AST on an industrial scale was first started in 1980 by Cyanotech Corporation (Kona, HI, USA) under the product name BioAstin® which is sourced from *H. pluvialis*, while in China, AST is obtained in the industry from by-product extracts of crustaceans and krill [24].

## 4. Astaxanthin Chemistry

Astaxanthin (AST) is a carotenoid with a 40-carbon tetraterpene consisting of linked isoprene units [31]. AST has the chemical formula C_40_H_52_O_4_, is a red carotenoid family, and belongs to the *xanthophylls* family with a molecular weight of 596,841 g/mol with names including 3,3′-dihydroxy-*β*, *β*′-carotene-4 or 4′-dione [32]. The red color in AST is determined by a system of 11 conjugated double bonds [24]. AST exists in esterified forms, free forms, stereoisomers, and geometric isomers.

**Figure 1:**
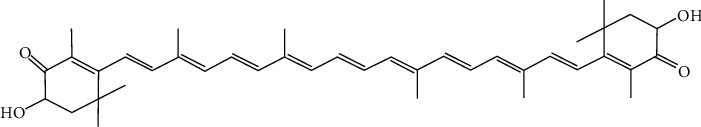
Chemical structure of astaxanthin.

AST consists of two terminal rings linked by a polyene chain. The AST molecule has two asymmetric carbon atoms located at the 3,3′ position on the *β*-ionone ring with a hydroxyl group (-OH) at both ends of the molecule [27]. The AST enantiomer depends on the hydroxyl groups attached to this carbon atom, which are named R,R (above the molecular plane), S,S (below the molecular plane), and R,S (meso form); AST which is synthesized from nature has a form different types, such as the *trans* (3S, 3′S)-isomer in *H. pluvialis* and (3R, 3′R)-isomer in yeast *X. dendrorhous*, whereas the synthetic AST type consisted of a mixture of optical isomers and meso forms (3R, 3′R), (3R, 3′S), and (3S, 3′S) which have a ratio of 1 : 2 : 1 [33]. Meanwhile, the AST stereoisomer could be found in krill (3R, 3′R) which contains the esterified form, while the (3S, 3′S) form could be found in salmon [34]. Thermodynamically, all-*trans* AST is more stable than the *cis* isomer but could undergo isomerization when exposed to light, heat, acids, and metal ions [35]. The chemical structure of astaxanthin is in Figure 1.

Esterification of AST could occur with different fatty acids, such as palmitic, oleic, stearic, or linoleic acid as a result of the presence of a benzoic hydroxyl group so that it could be resistant to temperature changes and phytochemical reactions compared to AST in its free form; in nature AST could be found in mono form as well as diesters, where diesters are more resistant to cold temperatures than the mono form and vice versa [24].

In addition, the antioxidative capacity of AST has been obtained due to the polar-nonpolar structure of AST which could contain hydrophobic polyene carbon chains in the lipid bilayer cell membrane, while the polar terminal ring is placed near its surface [36].

## 5. Bioavailability, Absorption, and Distribution of Astaxanthin

Animals could not synthesize carotenoids; hence, they must obtain these pigments from algae and plants [8]. The reddish color that is characterized by AST when mixed with the diet increases its glasses and luster in the feeder, which leads to the desire of birds to eat more quantities of the diet despite the conditions of heat stress, where the bird at heat stress compensates for the consumption of feed at night [37]. The quantity and rapidity of free-form AST being released from the conjugated form in feed affect the amount digested and absorbed [8]. Moreover, AST supplementation could induce a balance of intestinal flora, which could promote better absorption quality and aid in the degradation of high molecular weight proteins [38].

AST with the combination of fish oil promoted hypolipidemic/hypocholesterolemic effects in plasma and its increased phagocytic activity of activated neutrophils when compared with AST and fish oil alone [39]. Furthermore, AST is more polar than other carotenoids, which improves the extent and rate of absorption of being a soluble compound in the fat [40, 41]. Thanks to dietary lipids, AST could be dispersed in the digestive tract and dissolved in mixed micelles [42]. Then, AST is absorbed by the intestinal epithelial cells and directly delivered to the liver as portomicrons and thus incorporated into low-density lipoproteins (LDL) and very-low-density lipoproteins (VLDL) and transported to target tissues by the bloodstream [42]. AST could deposit in tissues such as the crest and egg yolk and in various tissues in the domestic chicken [8]. In addition, the distribution of AST in the tissues is influenced by various factors such as diet sources, sex, and animals strain.

## 6. Dietary Intake Levels of Astaxanthin

Astaxanthin from *P. rhodozyma* as much as 20 mg/kg could also increase the capacity of meat to retain water during the cooking process by keeping protein denaturation low, thereby reducing cooking loss, in which water loss in meat could reduce meat taste by maintaining meat juiciness [43]. However, the study conducted by Sun et al. [11] showed that WHC of the breast muscle was decreased by 80 mg AST/kg, and water deprivation reduces the nutrient value of meat by losing leachate and results in hardness and flavorless meat. These findings were similar to the effects of lower doses of AST supplementation on the WHC of muscles in the study conducted by Jeong and Kim, [8] using AST of 2.3 and 4.6 mg/kg.

In addition, dietary supplementation with AST-rich yeast, *Phaffia* rhodozyma (*Xanthophyllomyces dendrorhous*) 20 mg/kg, could soften the meat tested with SFV parameters which could occur due to the antioxidant properties of AST which has an activity to prevent myofibrillar protein oxidation which could harden meat due to the fact that it could promote protein cross-linking and aggregation [44]. AST also could increase the amino acid content in meat, especially glutamic acid (Glu), which could improve the taste of meat which occurs also due to antioxidant activity so that it could maintain protein degeneration and the formation of free amino acids [43].

On egg performance, research by Zhu et al. [45] showed that AST-rich *P. rhodozyma* could improve egg yolk color. Yolk discoloration is also found in other carotenoids [46]. Meanwhile, Walker et al. [47] revealed that egg yolk color could be improved by supplementing AST algae with palm toco. Yolk discoloration could occur due to the fact that AST could be directly stored in tissues without any biochemical modifications that are absorbed by animals [48]. While the egg yolk color comes from vitellogenin whose precursor is carotenoids [49], AST also could increase egg storage time by delaying the decrease in yolk index and yolk color [50]. However, Zhu et al. [45] showed that *P. rhodozyma* had no effect on shell thickness, Haugh unit (HU), shell strength, albumen height, and egg shape index. Meanwhile, the supplementation of AST from *Haematococcus* algae as much as 0.49%–0.47% which contains AST 0.35% and palm tocos into hens' diets did not affect performance or egg quality except for egg yolk color [47].

AST supplementation at 2.3 and 4.6 mg of AST/kg in the feed of *Phaffia* yeast linearly improved body weight and feed conversion ratio over the finisher and overall periods [8]. Ohh et al. [12] also reported that AST as much as 100 ppm of freeze-dried *Phaffia* yeast containing 5.0 mg/g plus 0.4% histamine could increase body weight by increasing consumption so that it is more efficient in growth. Moreover, AST powder from *H. pluvialis* at levels 10–40 mg/kg feed could improve feed conversion ratio, total weight gain, and final body weight average, with a significant decrease in the total mortality [37].

In contrast with the studies above, Sun et al. [11] reported that supplemental AST from *H. pluvialis* at 179 mg/kg or supplemental AST from *Phaffia* yeast at 22.5 mg/kg for 5 weeks did not affect the growth performance of chicks during the starter period. Other studies also reveal that dietary high-level inclusion of AST (213.4 mg/kg) from bioproduct *H. pluvialis* containing 2.84% AST has no adverse effect on the production performance of laying hens. In addition, AST supplementation at high doses is less effectively absorbed in egg yolk, thereby reducing egg yolk [42]. Dietary AST could improve meat quality and increase feed conversion ratio, total weight gain, and final body weight of broiler chicken in the administration of a small dose. However, in high-dose supplementation, it less significantly alters broiler performance.

## 7. Antioxidant Mechanism of Astaxanthin on Broilers

In terms of antioxidant activity, AST has stronger free radical scavenging activity against singlet oxygen than vitamin E, 10 times more potent than *β*-carotene, canthaxanthin, zeaxanthin, and lutein, 54 times stronger than *β*-carotene, 65 times more powerful than vitamin C, and 100 times more effective than *α*-tocopherol [51–54].

**Figure 2:**
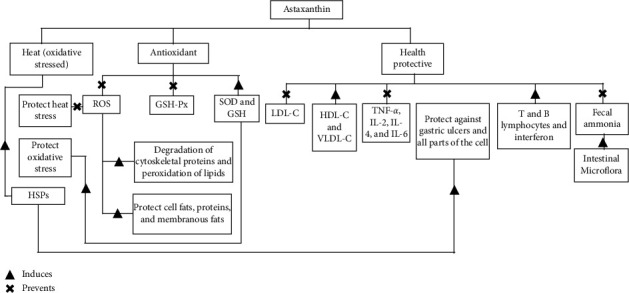
The role of astaxanthin in broiler chicken.

AST powder from *H. pluvialis* 10–30 mg/kg feed has a major role in protecting cells, fats, proteins, and membranous fats from oxidation by free radicals and removing all types of ROS (Figure 2) resulting from bird exposure to heat stress [37]. A previous study also showed that the DPPH and ABTS radical scavenging activity was 72–220 greater in the AST obtained from shrimp shell waste powder by 101.3 g than ascorbic acid [55].

The study by Gao et al. [25] showed that dietary AST derived from *H. pluvialis* which had an AST content of 1.3% as much as 50–100 mg/kg could increase the activities of SOD, T-AOC, and GSH and the total antioxidant capacity in the plasma, liver, and egg yolk. Meanwhile, in the study of Sun et al. [11], it was explained that DOC supplementation with AST from *H. pluvialis* (*H. pluvialis, Heliae, Gilbert, AZ*) at 10–80 mg/kg could increase GPX, GST, GSH, and GSSG after 3 weeks. However, it had no effect after 6 weeks of supplementation. In another study, supplementation with *H. pluvialis* containing AST 1.5% as much as 40 mg/kg of feed could increase SOD activity in the liver (361.42 U/mgprot) and in the kidneys (527.45 U/mgprot). In addition, GSH-Px in the liver showed a value of 25.34 U/mgprot and in the kidney showed a value of 32.83 U/mgprot and also could decrease MDA activity with an increase in AST intake [56]. Antioxidant status in the body is controlled by the production of ROS and supply of endogenous and exogenous antioxidants, and endogenous antioxidants including glutathione (GSH) and enzymes such as glutathione peroxidase (GSH-Px), glutathione reductase, and superoxide dismutase (SOD) play important roles in preventing oxidative stress [11, 57]. SOD could scavenge hydroxyl radicals and superoxide anions, whereas GSH-Px could reduce reactive oxygen species by promoting H_2_O_2_ decomposition [57, 58]. On the other hand, it remains unclear why the AST supplementations led to a dose-dependent decrease of GSH-Px activities in the thigh muscle of chicks [11].

In addition, in the study of Heng et al. [56], the expressions of nuclear factor E2-related factor 2 (NRF2) in the liver and kidneys were also increased. NRF2 could activate the expressions of antioxidant genes by nuclear translocation that occurs through the release of protein-1 associated with Kelch-like ECH and then transferred to the nucleus to bind to the adenylate uridylate-rich element sequences [59]. Other studies indicated that feeding a diet supplemented with freeze-dried *Phaffia* yeast containing 5.0 mg/g of AST and histamine was not very effective for the prevention of lipid peroxidation estimated by malondialdehyde (MDA) production, as measured by determining thiobarbituric acid reactive substances (TBARS) levels in the broiler, with liver SOD percentage of 67.7 ± 1.4 in liver and plasma TBARS percentage of 0.62 ± 0.08, both of which are biochemical indicators of the pathological states associated with oxidative stress and lipid peroxidation [12]. Grashorn [60] argues that some of the carotenoids consumed by poultry are used as antioxidants, while those that are not stored in the tissues or egg yolk will be broken down and excreted.

The antioxidant potential of AST is caused by a long carbon chain with conjugated double bonds and keto (C=O) moieties on each ionone ring [61]. The conjugated and -hydroxyketone double bonds in AST consist of a ketone group and a hydroxyl group at the end of the conjugated double chain, which could attract unpaired electrons from free radicals and donate electrons to free radicals [62]. In addition to having a hydrophilic and lipophilic structure, AST has a mechanism for scavenging oxygen free radicals and increasing the activity of antioxidant enzymes [25, 27].

## 8. Health Protective Effects of Astaxanthin on Broiler

The nutraceutical applications of AST previously reported include anticancer and antidiabetic applications and it has also been reported to have gastro-, hepato-, neuro-, cardio-, ocular, and skin-protective properties [63]. A study carried out by Awadh and Zangana [37] showed that AST from *H. pluvialis* as much as 10–40 mg/kg of feed could reduce the mortality ratio caused by the capacity of AST to enhance bird immunity by increasing the levels of T and B lymphocytes and the production of interleukin-producing immunoglobulins and interferon [64–66]. AST is also effective in increasing the proportion of microorganisms, especially probiotic bacteria originating from lactic acid bacteria in the intestine and spreading to the myosin fiber network which then covers the intestinal cells, thereby inhibiting pathogenic bacteria [13]. Suppression of the growth of Gram-negative bacteria could also protect against faecal ammonia which is a gas that is harmful to poultry [67].

However, AST had no effect on white blood cells, red blood cells, lymphocyte, and immunoglobulin G (IgG) amount, which possibly indicates that low AST concentrations such as 2.3 and 4.6 mg/kg of diet derived from fermented *P. rhodozyma* in a jar and then freeze-dried with the concentration of AST being 2,305 mg/kg of the medium, which may be insufficient to stimulate the immune system as compared with a higher concentration of 100 mg/kg of diet used [8, 68].

Astaxanthin (AST) also plays an essential role in the movement of cholesterol to high-density lipoprotein (HDL) [69]. Dietary AST supplementation of *H. pluvialis* containing 1.3% AST as much as 100 mg/kg significantly increased HDL-cholesterol (HDL-C) and very-low-density lipoprotein cholesterol (VLDL-C) and decreased low-density lipoprotein cholesterol (LDL-C) levels from 0.40 mmol/L to 0.20 mmol/L [25]. HDL and LDL are apolipoproteins that represent a form of lipid transport in the blood, where HDL could reduce the risk of cardiovascular disease [70, 71]. HDL could transport excess cholesterol from tissues and then back to the liver, while LDL could transport cholesterol from the liver to various organs in the body [72]. Meanwhile, VLDL-C is very important available for laying hens, which is a form of TG that is synthesized in the liver and then transported in the form of VLDL-C which will eventually be stored in the egg yolk [73].

However, Davinelli et al. [74] and Miyachi et al. [75] found that AST improved chronic inflammation. Research conducted by Dansou et al. [42] showed that immunomodulatory (IgM and IgG in serum) and anti-inflammatory activities were less present in a range of doses of 0.75 g/kg–7.5 g/kg microcapsules powder AstALPHYTM containing 2.84% of astaxanthin, which suggests that high doses of AST may be less efficient in modulating inflammation.

In addition, supplementation of 5 g esterified glucomannan (EGM)/kg diet, 10 mg/kg diet AST from *X. dendrorhous*, and their combination could partially or greatly alleviate the adverse effects induced by aflatoxin-B1- (AFB1-) contaminated feed, where AFB1-contaminated diets changed hematological and serum biochemical parameters, decreased liver antioxidant capacity, and resulted in hepatic injury in broilers [76].

## 9. Effect of Astaxanthin on the Heat (Oxidative Stress) Broilers

Exposing the broiler chicken to chronic high ambient temperatures would induce oxidative stress and inflammation, subsequently dysregulating their antioxidant defense, stress control, and lipid metabolism [77]. Heat stress induces the generation of ROS that impairs cellular structure and function [11, 78]. The excessive generation of ROS damages cell integrity through the degradation of cytoskeletal proteins and the peroxidation of lipids [79, 80].

A study by Hosseindoust et al. [15] showed that dietary AST supplementation from *Haematococcus* algae extraction was 40 and 80 mg/kg reduced corticosterone, which is also an indicator of chronic stress and will be increased during stress. In addition, there was also a decrease in gene expression of heat shock proteins (HSP27, HSP40, and HSP70) in the livers of broiler chicks; these three biomarkers could stabilize cytoskeletal proteins during heat stress by modulating oxidative and apoptotic activity. In addition, AST supplementation also support the role of HSP70 as a cellular thermometer effectively [81]. Expression of the HSP70 gene could protect against damage that occurs in cells during hot conditions by triggering protein expression [82]. The low HSP expression during AST supplementation may be due to the high antioxidant effect of AST [83]. Moreover, HSP stabilization of cytoskeletal proteins during heat stress occurs by upregulation of mitogen-activated protein kinases (MAPKs) such as P38MAPK and JNK, which in turn induces AKT1 [84].

## 10. Conclusion

Free radicals from heat stress rapidly initiate oxidative stress in broiler chicken, which is a major underlying cause of metabolic disorders and decreased broiler performance. AST has a potent antioxidant effect that is largely attributed to its unique chemical structure to protect all parts of the cell. To direct removal of free radicals and ROS, AST could regulate GSH and SOD to enhance the body's endogenous antioxidant defense system and also have the ability to maintain body cholesterol levels and levels of T and B lymphocytes. In addition, AST could improve broiler performance by reducing gene expression of heat shock proteins and also has a positive effect on the quality of poultry meat and eggs. Although several health-promoting effects of AST have been demonstrated, future studies are necessary for better understanding of the functions of AST.

## Figures and Tables

**Table 1:** Overview of recent application of astaxanthin in broiler chicken.

Animal	Dose rate	Major findings	References
Broiler	2.3–4.6 mg/kg in basal diet	Improved weight gain in the finisher period and linearly decreased feed conversion ratio in the finisher period	[8]
Broiler	50 mg/kg in basal diet	Improve in the average body weight and the weight increase and the amount of feed consumed, as well as an improvement in the food conversion rate	[2]
Broiler	10 and 20 mg/kg in basal diet	Improved T cell proliferation and IgG production	[13]
Broiler	Mixture of nanoselenium with astaxanthin at a concentration of 0.3 + 60 mg/kg	Increase body weight	[14]
Broiler	0.4% of histamine + 100 ppm of astaxanthin in basal diet	Astaxanthin did not affect histamine-dependent changes in chick body weight or weights of the gizzard and proventriculus	[12]
Broiler	40 or 80 mg/kg in basal diet	Decreased the hyperthermic stress level and improved meat quality, as well as antioxidant status of chickens exposed to heat stress	[15]
Broiler	Basal diet supplemented with 0.15% astaxanthin	Increased both the redness and yellowness of skeletal muscle and decreased the muscle MDA concentration	[16]
Broiler	Diet containing 100 ppm astaxanthin	Did not show anti-inflammatory effects in chickens	[17]
Broiler	10–80 mg/kg in basal diet	Affected the hepatic gene expression and protein production related to redox status, heat stress and inflammation, and lipid metabolism	[11]
Broiler	7.5% of shrimp waste flour in broiler diets	Improve carcass weight, carcass percentage, abdominal fat of broilers	[18]
Broiler	10 and 20% silage of rainbow trout in basal diet	Improve feed consumption, live weight gain, feed conversion index, mortality, carcass yield, economic conversion index, and economic profitability index	[19]
Broiler	2% of shellfish processing industry waste slurry in broiler diets	Improved body weight, weight gain, feed intake, and FCR	[20]

## Data Availability

The research data are presented in tables, diagrams, and graphs in the articles. Supportive data for discussion and comparison were from previous studies which have been cited from recent journal related to the focus of this article. These data are publicly available and accessible online. Detailed sources are provided in References of the manuscript.

## Additional Points

(1) The objective of this review was to collect and critically discuss the information available on the use of astaxanthin as a potential antioxidant to improve health and production performance of broiler chicken. An electronic search was conducted using keywords germane to the topic to identify relevant studies. The publication characteristics, study design, study conduct and reporting, and study relevance were used as criteria for eligibility. An author index compiled all articles that met the inclusion criteria. The keywords used for the search were astaxanthin, antioxidant, broiler chicken performance, health protective, and oxidative stress. The full-text articles published from 2000 to 2021 were combined from the search results and duplicates were removed. Information from each selected source was compiled while accounting for the strengths and weaknesses of each article. Studies that were weak in subject number or contained evident biases were considered but were not contributive in summary. (2) By analyzing the methodologies of the articles, the most commonly used astaxanthin was critically assessed for its role in reducing free radicals and oxidative stress, helping to maintain a healthy state, and improving the performance of broiler chickens by increasing the daily feed intake, followed by improvement in the food conversion rate. It is revised in this paper.
